# Supplementary figures and images for: Interactions of Segmented Filamentous Bacteria (Candidatus Savagella) and bacterial drivers in colitis-associated colorectal cancer development
Source: PLoS One. 2020 Jul 24;15(7):e0236595. doi: 10.1371/journal.pone.0236595 (PMC7380633; doi:10.1371/journal.pone.0236595)

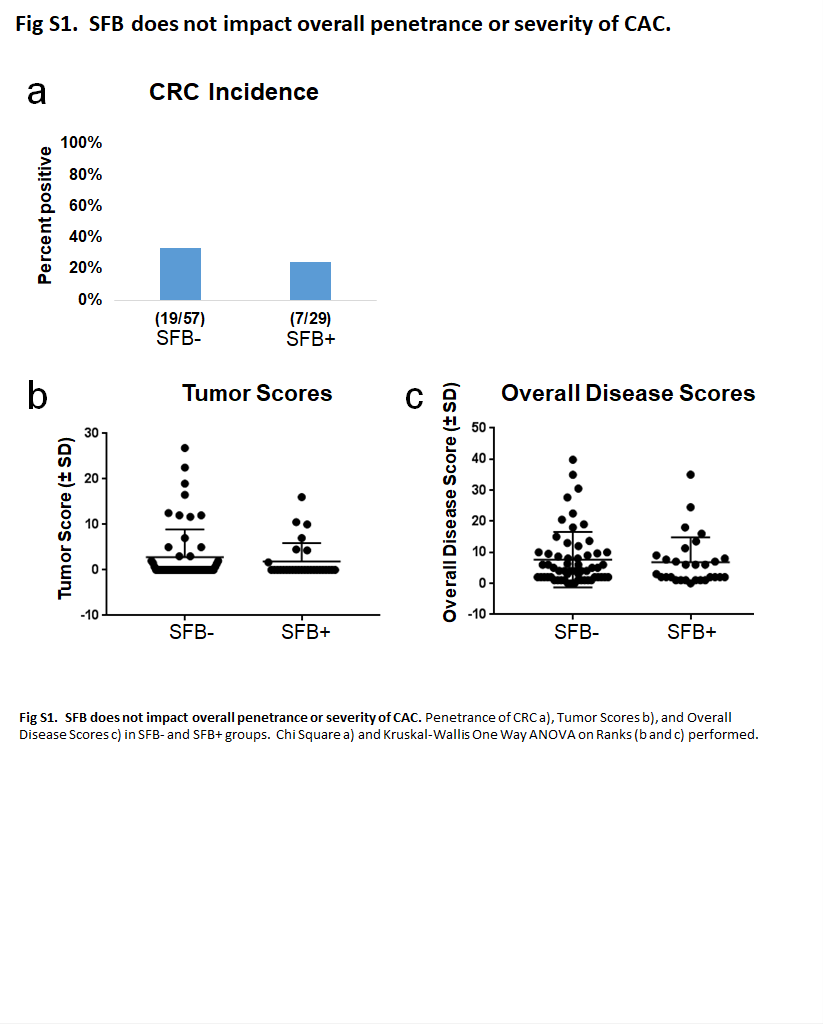

Supplement: S1 Fig — Penetrance of CRC a), Tumor Scores b), and Overall Disease Scores c) in SFB- and SFB+ groups. Chi Square a) and Kruskal-Wallis One Way ANOVA on Ranks (b and c) performed. (TIF) [file pone.0236595.s001.tif]

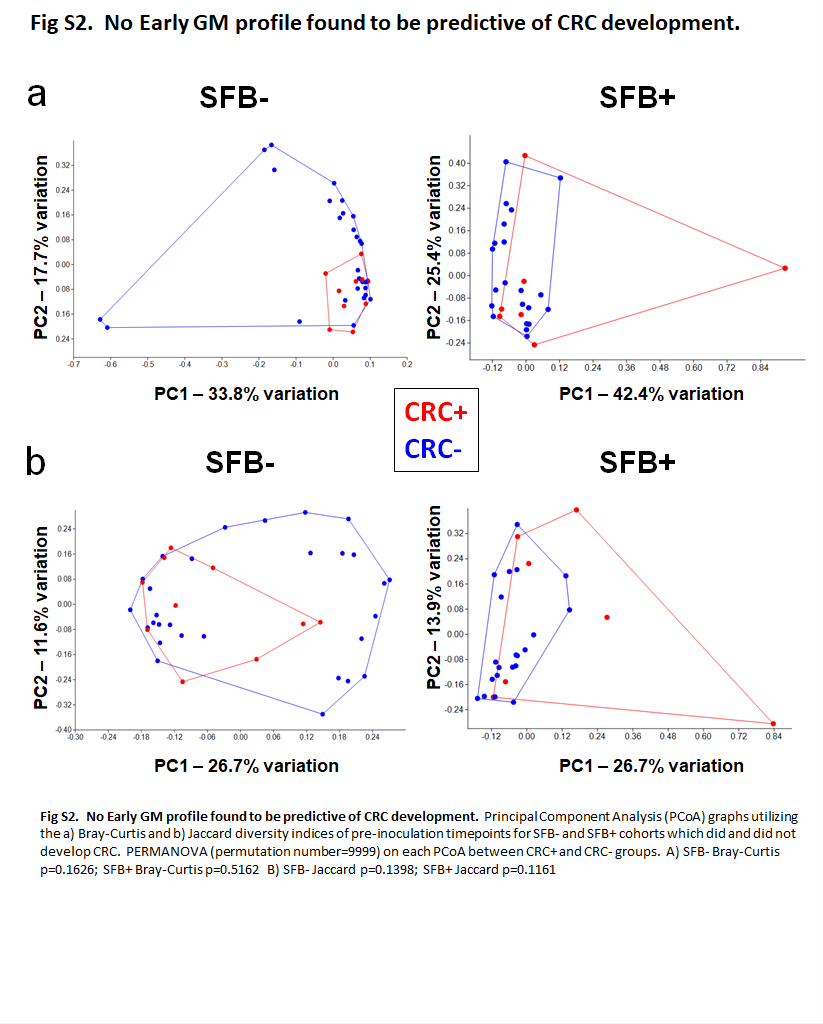

Supplement: S2 Fig — Principal Component Analysis (PCoA) graphs utilizing the a) Bray-Curtis and b) Jaccard diversity indices of pre-inoculation timepoints for SFB- and SFB+ cohorts which did and did not develop CRC. PERMANOVA (permutation number = 9999) on each PCoA between CRC+ and CRC- groups. A) SFB- Bray-Curtis p = 0.1626; SFB+ Bray-Curtis p = 0.5162 B) SFB- Jaccard p = 0.1398; SFB+ Jaccard p = 0.1161. (TIF) [file pone.0236595.s002.tif]

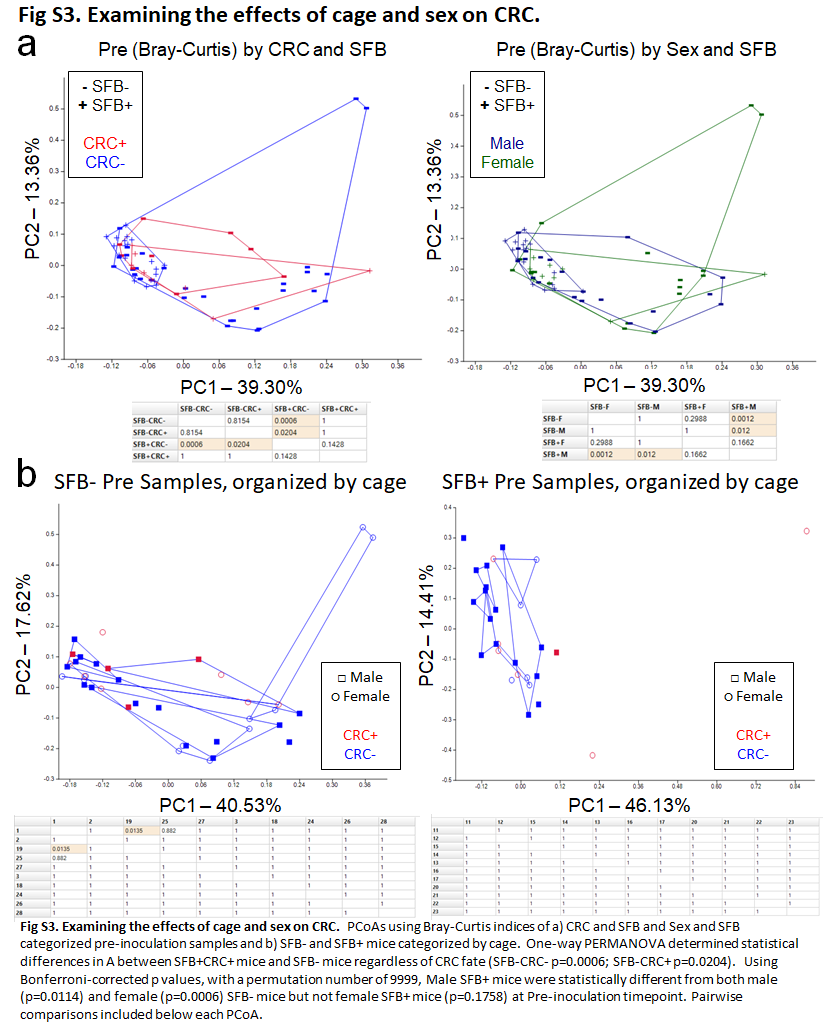

Supplement: S3 Fig — PCoAs using Bray-Curtis indices of a) CRC and SFB and Sex and SFB categorized pre-inoculation samples and b) SFB- and SFB+ mice categorized by cage. One-way PERMANOVA determined statistical differences in A between SFB+CRC+ mice and SFB- mice regardless of CRC fate (SFB-CRC- p = 0.0006; SFB-CRC+ p = 0.0204). Using Bonferroni-corrected p values, with a permutation number of 9999, Male SFB+ mice were statistically different from both male (p = 0.0114) and female (p = 0.0006) SFB- mice but not female SFB+ mice (p = 0.1758) at Pre-inoculation timepoint. Pairwise comparisons included below each PCoA. (TIF) [file pone.0236595.s003.tif]

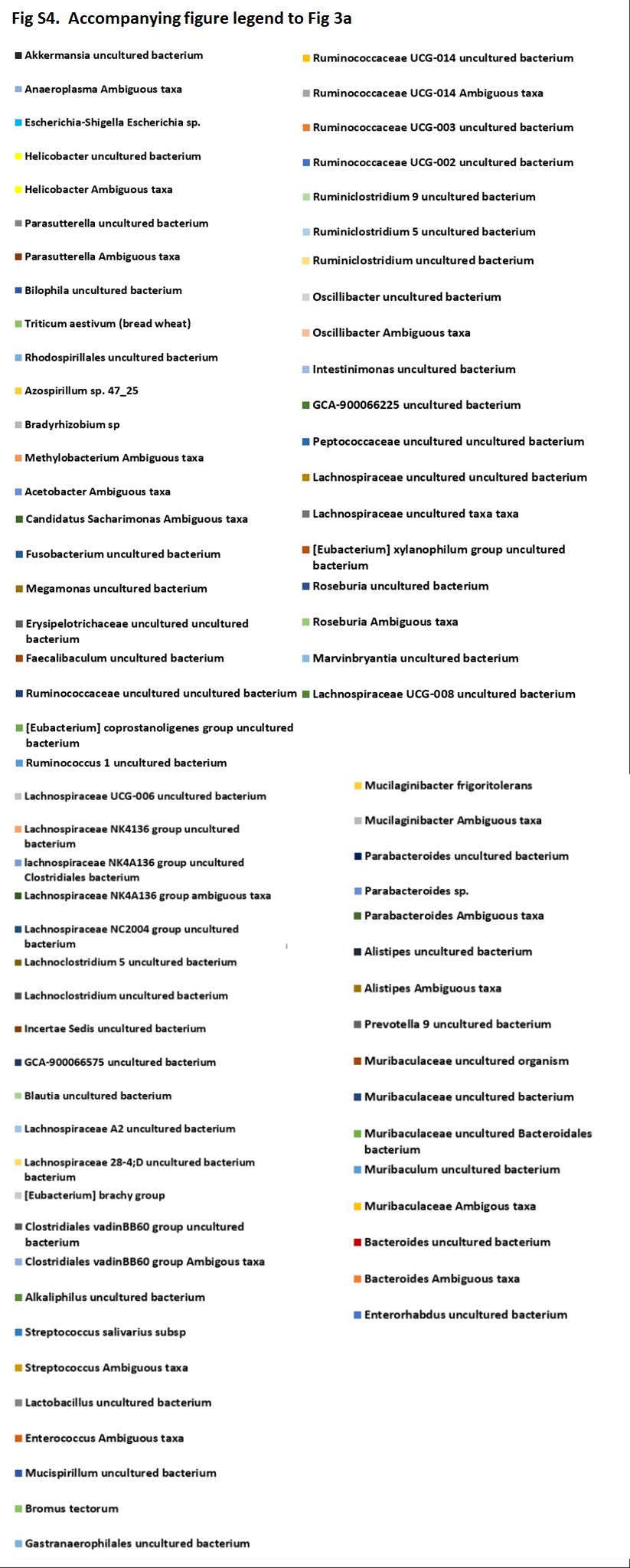

Supplement: S4 Fig — Corresponding OTUs to each segment of the barcharts shown on Fig 3A. (TIF) [file pone.0236595.s004.tif]

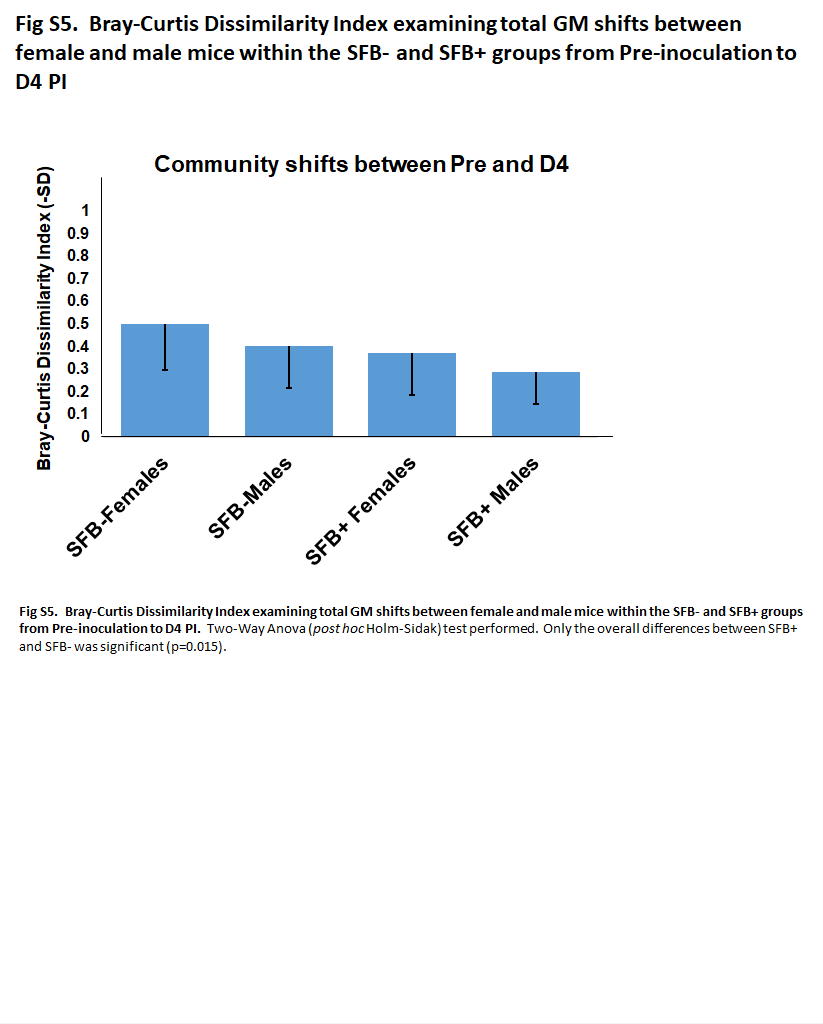

Supplement: S5 Fig — Two-Way Anova (post hoc Holm-Sidak) test performed. Only the overall differences between SFB+ and SFB- was significant (p = 0.015). (TIF) [file pone.0236595.s005.tif]

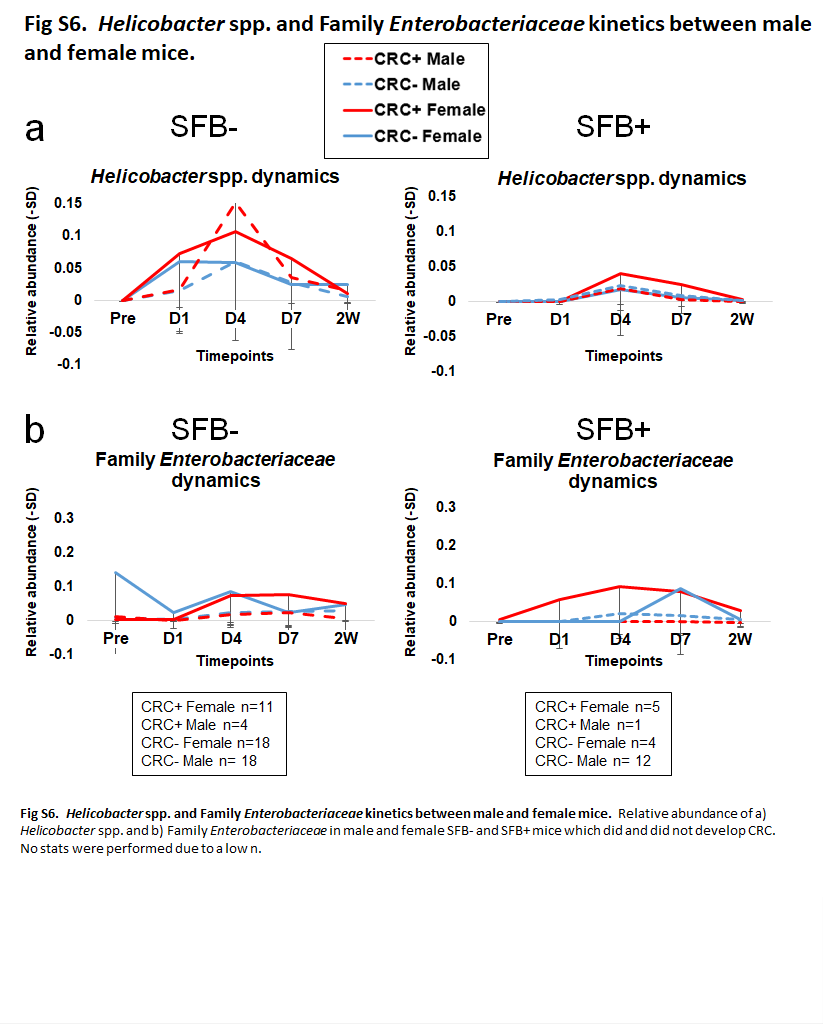

Supplement: S6 Fig — Relative abundance of a) Helicobacter spp. and b) Family Enterobacteriaceae in male and female SFB- and SFB+ mice which did and did not develop CRC. No stats were performed due to a low n. (TIF) [file pone.0236595.s006.tif]

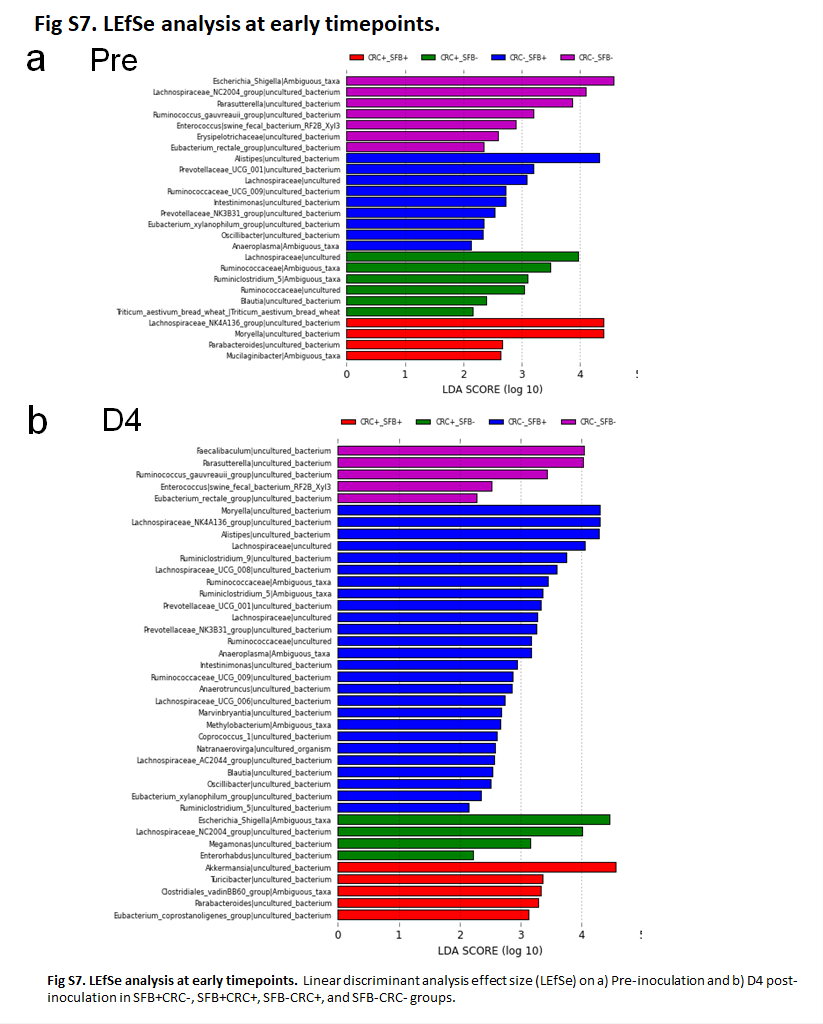

Supplement: S7 Fig — Linear discriminant analysis effect size (LEfSe) on a) Pre-inoculation and b) D4 post-inoculation in SFB+CRC-, SFB+CRC+, SFB-CRC+, and SFB-CRC- groups. (TIF) [file pone.0236595.s007.tif]
